# Acute kidney injury after elective adult cardiac surgery

**DOI:** 10.1186/cc13563

**Published:** 2014-03-17

**Authors:** A Darbar, G Lau, R Porter

**Affiliations:** 1 Glenfield Hospital, Leicester, UK

## Introduction

Acute kidney injury (AKI) is a significant complication following cardiac surgery associated with an increase in morbidity, hospital stay and mortality. Although the estimated incidence of AKI following cardiac surgery is 30%, few studies have identified the incidence of AKI following cardiac surgery as defined by the Kidney Disease: Improving Global Outcomes (KDIGO) group [1]. We conducted a prospective observational study at our institution to identify the incidence and staging of AKI according to the KDIGO definition. We also aim to identify the factors that predispose adult patients to developing AKI post cardiac surgery.

## Methods

A prospective analysis was performed on 103 adult patients admitted to ICU post-cardiac surgery from September to October 2013. Data for perioperative risk factors and renal biochemical markers were collected up to the sixth postoperative day and are expressed as mean (SD).

## Results

Ordered logistic regression was used to analyse the data. Thirty- three per cent of cardiac surgery patients at our institution developed AKI. Factors such as poor left ventricular (LV) function, low preoperative haematocrit and low preoperative glomerular filtration rate (GFR) were significant risk factors (*P *= 0.03, *P *= 0.04 and *P *< 0.01 respectively) for developing postoperative AKI following cardiac surgery. See Table 1.

**Table 1:** Results

Age (years)	66 (13.8)
Male	69
Female	34
No AKI	69
KDIGO 1	22
KDIGO 2	6
KDIGO 3	6

## Conclusion

Our results suggest that we should focus on LV function and GFR as predictors for developing AKI following cardiac surgery. Strategies to increase preoperative haematocrit should be investigated to reduce in the incidence and severity of postoperative AKI.
